# Supplementary material for: The Primary Sclerosing Cholangitis (PSC) Wellbeing Study: Understanding psychological distress in those living with PSC and those who support them
Source: PLoS One. 2020 Jul 6;15(7):e0234624. doi: 10.1371/journal.pone.0234624 (PMC7337345; doi:10.1371/journal.pone.0234624)
Supplement: Attachment — Submitted filename: Response to reviewers_The PSC Wellbeing Study.docx [file pone.0234624.s001.docx]

| **Comments from Editorial Office:** | **Researchers’ responses:** |
| --- | --- |
| Please ensure that your manuscript meets PLOS ONE's style requirements, including those for file naming. | We thank the editorial office for bringing this to our attention and have not amended the article to fit with styling requirements. |
| We note you have included a table to which you do not refer in the text of your manuscript. Please ensure that you refer to Table 1 in your text; if accepted, production will need this reference to link the reader to the Table. | Thank you for bringing it to our attention. The table (now renamed as Table 2) has now been cited in the final sentence of Paragraph 1 of our results. |
| The wider readership of the journal will benefit from further information on PSC in the introduction. Some context on how this condition could be unique or of significance in comparison to other progressive conditions and particularly in relation to psychological distress should be added. | We agree with the editorial office’s suggestion and have added the following paragraph to the introduction:  *“Primary Sclerosing Cholangitis (PSC) is a cholestatic illness caused by inflammation and scarring of the liver’s bile ducts.^1^ Although some individuals may be asymptomatic at first, the illness is associated with burdening symptoms such as itching, fatigue, jaundice and abdominal pain.^1,2^ It is prevalent in 0-16.2 cases per every 100,000 individuals, though prevalence rates appear to be on the increase.^1,3^ At present there is no known cause or medical treatment to halt or slow the progression of the illness, with some of those diagnosed with PSC progressing to end-stage liver disease that requires transplantation or developing cancer. ^4,5^”* |
| Have the authors made all data underlying the findings in their manuscript fully available | We understand and agree that all data, particularly if quantitative in nature, should normally be fully available without restriction. However, we cannot share the coding and analysis of the data as this would breach participants’ confidentiality. |
| **Comments from reviewer 1:** | **Researchers’ responses:** |
| 1. It would round up the introduction and would be very useful to the readers not very familiar with PSC to describe what it exactly is, the course of the disease and symptoms and its prevalence. Otherwise the scope of the study is well described in the introduction. | We agree with the reviewer’s suggestion and have added the following paragraph to the introduction:  *“Primary Sclerosing Cholangitis (PSC) is a cholestatic illness caused by inflammation and scarring of the liver’s bile ducts.^1^ Although some individuals may be asymptomatic at first, the illness is associated with burdening symptoms such as itching, fatigue, jaundice and abdominal pain.^1,2^ It is prevalent in 0-16.2 cases per every 100,000 individuals, though prevalence rates appear to be on the increase.^1,3^ At present there is no known cause or cure for the illness, with some cases progressing to end-stage liver disease that requires transplantation. ^4^”* |
| 2. Please add a table describing the sample; sociodemographic data and characteristics related to the disease. I guess you need to do this separately for patients and for relatives. | We thank the reviewer for this comment, however, feel that this request was already addressed as the information was provided in the participants section of the article. We regret that we do not have further detail on the participants. |
| 3. Please describe the methods of data collection Asynchronous Virtual Focus Groups and give a reference if possible | We have included the following sentences in the recruitment section of the method:  *“AVFGs are online focus groups where individuals log onto an online platform to post responses to questions.^6,7^ Participants are typically asked to write one or more responses to each question within a given time limit.”* |
| 4. The “interview guideline”/questions used by the researchers for the Focus groups are missing. Please add a Table or paragraph with this information | We thank the reviewer for this comment and have added a Table (now Table 1) with the list of questions asked. |
| 5. Please explain how you developed the questionnaire/questions. | We have now included the following sentences in the procedure section of the method:  *“These topics were identified by members of PSC Support, who had lived experience of PSC. For a list of questions, please see Table 1 “* |
| 6. It would be important to know the extent/size of the material/text that was analysed. How many pages? Words? Also where there any conflicts in communication or interaction? Did the researchers/moderators have to intervene? And if yes, why and how? And how could this have affected the data? | 37,602 words from three groups of patients answering the four questions were analysed. 10,976 words from one group of caregivers answering the four questions were analysed.  The researchers did not have to intervene in the online discussions as participants were very respectful of each other’s opinions and experiences. The researchers, on occasion, added a few probing questions to some participants’ answers for clarification, in the same manner as an in-person focus group. The researchers also prompted participants by email to remind them of how much time was left to respond to the week’s question, typically on Fridays ahead of Monday’s question switch. |
| 7. Regarding the data analysis you mention “thematic narrative analysis” and even though you describe it, it remains a bit vague and there is no reference regarding this method of analysis. Please add a reference. | We have now included references. |
| 8. You present the sample demographics in the results. I would suggest putting part this in methods. | This data has now been placed in the method section. |
| 9. The results are clearly written, there is a cohesive narrative, it is captivating to read and the text passages add value to the narrative. Nevertheless, I feel the way you have presented the results in terms of “chronological” order (“living with PSC, diagnosis, life after diagnosis, preparing of transplantation, life after transplant) helps the overall narrative by creating an overall thread, but does not do justice to very insightful findings and the richness of the results, that seem to go under in the general chronological categories. A solution to this would be to either add the subcategories of your analysis in the text or highlight (bold or cursive) the subcategories (e.g. grief, lack of support, problematic communication with physicians) within the text. In this way the content of the findings would become more clear and could function as a take home message to the reader. | We thank the reviewer for this suggestion and have added subcategories within the results section to clarify and break up sections into smaller components. |
| 10. The section “life after transplant” is very thin compared to the rest of the paper. | We agree that this section is quite short. We did not specifically ask participants about life after transplant as we were mindful that only very few participants experienced transplant. The little detail provided in this section pertains to information which the participants shared of their own volition and which we did not wish to waste, particularly as so little is known about the experiences of this population. We have added the following sentences to the discussion to explain this:  *“Furthermore, we posit that saturation was unlikely to have been reached with regard to caregivers’ accounts, and narratives regarding transplantation. Participants were not specifically asked about transplantation and fewer caregivers contacted the research team to participate. This information was included in light of the limited available research on these topics. Nonetheless, we urge readers to interpret those particular segments with caution.”* |
| 11. Table 1 is interesting but it appears in the text out of the blue. You need to embed it and explain this better | Thank you for bringing it to our attention. The table has now been cited in the final sentence of Paragraph 1 of our results, as it was describe here. |
| 12. The discussion is very short and weak and it needs to be elaborated. It is basically just a paragraph summarising the findings arguing they cannot be discussed as there have been no previous similar studies to compare. I beg to differ regarding this. I think basically almost each of the subcategories of the results/analysis can be discussed e.g. in relation to other literature (if not studies regarding PSC, it can be studies of similar patient groups); or in relation to weaknesses of the health care system; the attitude of professionals; the role of the support system; the relatives support etc. | We thank the reviewers for this insightful comment and have added the following paragraph to the discussion:  *“Although there is dearth of literature that documents the experiences of those living with PSC and their caregivers, many of the findings of this study are comparable to the experiences of those with other rare, serious or progressive illnesses. For instance, the presence of psychological distress is notable in those living with rare or advanced cancers.^8,9^ Reduced physical functioning due to disabling symptoms, and reduced wellbeing and functioning in psychological, work and social spheres have been reported in those living with other illnesses.^10^ Shock at receiving a diagnosis of an incurable illness and feeling distressed about the progressive nature of it is recognised in the narratives of those living with incurable cancer.^11^ Participants’ sense of isolation and loneliness is also reflected in the accounts of those living with rare, incurable or painful illness.^12-14^ Finally, coping responses such as denial, acceptance, positivity and slowing down the pace of life were reported by individuals who, like many of those living with PSC, had multiple chronic conditions or living with incurable cancer.^11,15^”* |
| 13. You can remove the heading “summary of findings from the discussion”; It does not add anything | This has now been omitted. |
| **Comments from reviewer 2:** | **Researchers’ responses:** |
| 1. It would be advisable to state the epistemological approach in the abstract and methods section of the manuscript clearly, whether it was interpretative or phenomenological. Based on the framework mapping and style of presentation it comes across as phenomenological. However, it needs to be specified and justified. | We thank the reviewer for this comment. Our research did indeed take a phenomenological approach as it was interested in understanding the subjective experiences of individual participants and the meaning that participants attributed to these experiences. We have now included this in the manuscript. |
| 2. Introduction: Given the rarity of this condition, it may be useful to add some epidemiological information on PSC. For example, how common it is (prevalence) in the UK or globally and why it may be important to identify sources of distress in this population. | We agree with the reviewer’s suggestion and have added the following paragraph to the introduction:  *“Primary Sclerosing Cholangitis (PSC) is a cholestatic illness caused by inflammation and scarring of the liver’s bile ducts.^1^ Although some individuals may be asymptomatic at first, the illness is associated with burdening symptoms such as itching, fatigue, jaundice and abdominal pain.^1,2^ It is prevalent in 0-16.2 cases per every 100,000 individuals, though prevalence rates appear to be on the increase.^1,3^ At present there is no known cause or cure for the illness, with some cases progressing to end-stage liver disease that requires transplantation. ^4^”* |
| 3. Methodology: It is generally well described. But I had several unanswered questions as I read through the content - Interview guide / weekly questions asked to the participants should be included in the manuscript | We thank the reviewer for this comment and have added a Table (now Table 1) with the list of questions asked. |
| - Participants – what stage of the condition were they in? It will be useful to add this to understand some context to the discourse. It is also mentioned that most were picked up during scans for another serious condition. How was it ensured that the content related only to PSC and not for other similar/ more serious conditions? | We appreciate the usefulness of this information, however, we did not collect data pertaining to stage of illness. This has now been included as a limitation of the study.  Though we asked participants to describe their experiences of PSC, it is possible that their experiences of psychological distress may have been impacted by living with comorbidities. PSC came as an additional burden to many who were already living with other conditions and we didn’t think it was useful to separate |
| - Only 3 participants had experience with transplant. How was this discussion conducted in a group with the rest? Was there adequate saturation in the content? | We agree that this section is quite short. We did not specifically ask participants about life after transplant as we were mindful that only very few participants experienced transplant. The little detail provided in this section pertains to information which the participants shared of their own volition and which we did not wish to waste, particularly as so little is known about the experiences of this population. We have added the following sentences to the discussion to explain this:  *“Furthermore, we posit that saturation was unlikely to have been reached with regard to caregivers’ accounts, and narratives regarding transplantation. Participants were not specifically asked about transplantation and fewer caregivers contacted the research team to participate. This information was included in light of the limited available research on these topics. Nonetheless, we urge readers to interpret those particular segments with caution.”* |
| - Authors should comment on the overall assessment of data or thematic saturation in the manuscript. | We agree with the reviewer and have added the following sentences to the discussion:  *“This study had four main strengths and three limitations. The use of online focus groups allowed the researchers to access geographically diverse populations within the UK who may have otherwise been unable to participate in the study using conventional qualitative methods. It also allowed participants, who could be very symptomatic, to take part in the study from the comfort of their own home by logging in at a time of their choosing. Such ease of access may have facilitated the researchers in reaching saturation, whereby no new knowledge was emerging. Participants also wrote their own narratives, which may have provided a potentially therapeutic element and eliminated the need for transcription. However, participation in the study was literacy and tech-literacy dependent. Participants also responded to advertisements in order to participate in the study which may have introduced a self-selection bias in our sample. Furthermore, we posit that saturation was unlikely to have been reached with regard to caregivers’ accounts, and narratives regarding transplantation. Participants were not specifically asked about transplantation and fewer caregivers contacted the research team to participate. This information was included in light of the limited available research on these topics. Nonetheless, we urge readers to interpret those particular segments with caution.”* |
| - I am not sure about the inclusion of caregivers in this work – only 8 carers were included. Did they participate in the same forum/discussion platform? I do not believe this number would be adequate to reach saturation. Were the caregivers relatives of the patient-participants and if so, were there any duplicity issues in the discussions? how were these assessed and addressed? Given the length of the manuscript and the lack of saturation, I would like the authors to consider focusing only on patients. Or compressing the content from caregivers in very brief 1-2 paragraphs (highlighting the lack of saturation as a major limitation in the discussion) | Three of the four focus groups comprised of individuals living with PSC, and one comprised of caregivers. One caregiver in our study was the relative of one of the patients in our study. We agree with the reviewer that saturation is a likely issue in this group, however, in light of the lack of available literature on this group, we feel it would be more helpful to include some information about this group whilst highlighting saturation as a limitation. |
| - Elaborate the role of the moderators during the discussions. How was adequate participation from all discussants ensured? Did they participate in the discussions? | The researchers did not have to intervene in the online discussions as participants were very respectful of each other’s opinions and experiences. The researchers, on occasion, added a few probing questions to some participants’ answers for clarification, in the same manner as an in-person focus group. The researchers also prompted participants by email to remind them of how much time was left to respond to the week’s question, typically on Fridays ahead of Monday’s question switch.  As with in-person focus groups, some participants provided more input than others. In a minority of cases, where we noticed that some participants did not yet comment after approximately 5 days of the question being posted onto the online platform, we contacted the participants to remind them of the study. Due to the nature of PSC, we felt it was kindest to contact them privately by email in case they experienced acute symptoms. |
| 4. Results: Although well described, results section is rather long. It may be beneficial to provide a quick summary or schematic representation of the themes and subthemes. Also useful to add the age of the participants attributed to the verbatim quotes. | We agree with the reviewer and have attached a table (Table 3) outlining the themes according to participant type and timeline. Ages have also been added to the quote attributions. |
| Some content needs rephrasing for further clarification: - “However, such groups also had the potential to scare or disturb participants when younger or more advanced cases of PSC were discussed” - provide a quote to explain it | We have now included a quote to illustrate this:  *“The Facebook group does have it’s down side because it makes you very aware of all the ‘bad stuff’ and you watch people suffering and going through hell, it is particularly disturbing to watch the suffering of the younger members of the group.”* |
| - “Both disparities in NHS budget allocations across the UK, and poor collaboration and communication across specialist teams and primary care meant advocating to receive appropriate medical attention and support was a common occurrence, both in secondary and primary care.” - ‘disparities in NHS budget allocations across the UK’ – were these discussed by the participants? Good to provide 1-2 quote to explain it.  - “These questioned whether physicians understood the psychological implications of living with PSC.”- this is not clear. Please check. | We have now included the following quotes:  *“The different funding pots of the NHS have a real influence on quality of care – [retracted hospital name] were surprised that my GP was prepared to prescribe one of my anti rejection drugs, as their experience was that many wouldn't. As a patient you shouldn't get caught up in these inter-NHS issues.”*  *“I fear that sometimes it comes down to who’s budget is paying for the tests or medication. I know my hospital stopped giving me medication at the hospital and told me to go to my GP, this was so that the cost didn’t come from the hospital budget but from the GPs.”*  *We have also included the following sentence for clarification: “Not being asked about their psychological wellbeing, led those living with PSC to question whether physicians fully understood the psychological impact of the illness.”* |
| - “The first specialist I saw had no grasp at all what it is like to be told you have a disease that is chronic and has no cure. He was very blasé and to quote him ‘well you're a big fella so I thought you wouldn't have any issues, I am surprised you took it this way’.” BB, male – Should this be part of the ‘experience of diagnosis’ theme? | This quote referred to a discussion between a specialist and individual living with PSC at a separate appointment following diagnosis, however, considering the proximity of this discussion to the time of diagnosis, we agree that it could be more helpful to the reader if it was place under the ‘experience of diagnosis’ theme. |
| In addition, as stated previously, please re-assess or justify the thematic analysis around transplantation. It is not clear to whom these experiences relates to. | We agree that this section is quite short. We did not specifically ask participants about life after transplant as we were mindful that only very few participants experienced transplant. The little detail provided in this section pertains to information which the participants shared of their own volition and which we did not wish to waste, particularly as so little is known about the experiences of this population. We have added the following sentences to the discussion to explain this:  *“Furthermore, we posit that saturation was unlikely to have been reached with regard to caregivers’ accounts, and narratives regarding transplantation. Participants were not specifically asked about transplantation and fewer caregivers contacted the research team to participate. This information was included in light of the limited available research on these topics. Nonetheless, we urge readers to interpret those particular segments with caution.”* |
| Likewise, under-saturation of ‘Support’ theme needs to be carefully considered. Possibly significantly reduce the content and remove the theme on ‘support through transplantation’ (only 1 carer experience) | We thank the reviewer for this comment and agree that this section does not add to the overall article considering the lack of saturation on the topic. |
| Indicate the reference to Table 1. | Thank you for bringing it to our attention. The table has now been cited in the final sentence of Paragraph 1 of our results. |
| 5. Discussion: Needs further depth and thought. Much of the discussion is a repetition of the results. Although literature in PSC could be limited, authors should draw some parallels with other severe conditions. I gather psychological distress is universal in all severe health conditions, be it cancer, dementia or other GI conditions. It is important to relate it to the study aims and highlight the relevance and contrition of this work in the purview of PSC care and management. Authors provide some recommendations, whoever these could be possibly strengthened by drawing upon results from other studies in similar conditions. | We thank the reviewers for this insightful comment and have extended the discussion. We have also added the following paragraph to the discussion in relation to the literature:  *“Although there is dearth of literature that documents the experiences of those living with PSC and their caregivers, many of the findings of this study are comparable to the experiences of those with other rare, serious or progressive illnesses. For instance, the presence of psychological distress is notable in those living with rare or advanced cancers.^8,9^ Reduced physical functioning due to disabling symptoms, and reduced wellbeing and functioning in psychological, work and social spheres have been reported in those living with other illnesses.^10^ Shock at receiving a diagnosis of an incurable illness and feeling distressed about the progressive nature of it is recognised in the narratives of those living with incurable cancer.^11^ Participants’ sense of isolation and loneliness is also reflected in the accounts of those living with rare, incurable or painful illness.^12-14^ Finally, coping responses such as denial, acceptance, positivity and slowing down the pace of life were reported by individuals who, like many of those living with PSC, had multiple chronic conditions or living with incurable cancer.^11,15^”* |
| Minor corrections: Correct the second sentence in the abstract. It should be “a recent scoping review by the authors..” Last sentence in the introduction: Change to past tense. “One participant wrote that their GP told them that it was the patient’s responsibility patient to ‘chase’ the consultant” – delete patient after responsibility | Thank you, we have now made those corrections. |
