# Supplementary material for: The Primary Sclerosing Cholangitis (PSC) Wellbeing Study: Understanding psychological distress in those living with PSC and those who support them
Source: PLoS One. 2020 Jul 6;15(7):e0234624. doi: 10.1371/journal.pone.0234624 (PMC7337345; doi:10.1371/journal.pone.0234624)
Supplement: Attachment — Submitted filename: Response to reviewer 2_The PSC Wellbeing Study.docx [file pone.0234624.s002.docx]

| **Comments from reviewer 2:** | **Researchers’ responses:** |
| --- | --- |
| Thanks for addressing the comments and suggestions carefully. Research in this area is rare and its useful to understand PSC sufferers' experiences. I have one additional suggestion - authors could look at the limitations section carefully and elaborate it a bit further based on some of my and other reviewer's comments that couldn't be addressed due to lack of data. Rest of the manuscript reads well. | We thank the reviewer for this comment. We have expanded and added to our limitations as follows:  *“Strengths and weaknesses*  *This study had four main strengths and four limitations. The use of online focus groups allowed the researchers to access geographically diverse populations within the UK who may have otherwise been unable to participate in the study using conventional qualitative methods. It also allowed participants, who could be very symptomatic, to take part in the study from the comfort of their own home by logging in at a time of their choosing. Such ease of access may have facilitated the researchers in reaching saturation, whereby no new knowledge was emerging. Participants also wrote their own narratives, which was potentially therapeutic in itself and also eliminated the need for transcription. However, the use of this method also reduced participants’ spontaneity of speech as these could edit their text prior to submitting it. Participation in the study was literacy and tech-literacy dependent which may have excluded some of our target population. Participants also responded to advertisements in order to participate in the study which may have introduced a self-selection bias in our sample. Furthermore, we posit that saturation was unlikely to have been reached with regard to caregivers’ accounts, and narratives regarding transplantation. Participants were not specifically asked about transplantation and fewer caregivers contacted the research team to participate. This information was included in light of the limited available research on these topics, therefore, must be interpreted with caution.”* |
